# Advances of Osteosarcoma Models for Drug Discovery and Precision Medicine

**DOI:** 10.3390/biom13091362

**Published:** 2023-09-07

**Authors:** Linyun Tan, Yitian Wang, Xin Hu, Guifeng Du, Xiaodi Tang, Li Min

**Affiliations:** 1 Department of Orthopedic Surgery and Orthopedic Research Institute, West China Hospital, Sichuan University, Chengdu 610064, China; tanlinyun@stu.scu.edu.cn (L.T.); wangytbone1991@163.com (Y.W.); huxin@stu.scu.edu.cn (X.H.); duguifeng@stu.scu.edu.cn (G.D.); tangxd@stu.scu.edu.cn (X.T.); 2 Department of Model Worker and Innovative Craftsman, West China Hospital, Sichuan University, Chengdu 610064, China

**Keywords:** osteosarcoma models, cell lines, 3D culture technology, mice models, canine models, drug discovery

## Abstract

The management of osteosarcoma (OS) patients presents a significant clinical challenge. Despite progress in conventional and targeted therapies, the survival rate of OS patients remains limited largely due to therapy resistance and the high metastatic potential of the disease. OS models that accurately reflect the fundamental characteristics are vital to the innovation and validation of effective therapies. This review provides an insight into the advances and challenges in OS drug development, focusing on various preclinical models, including cell lines, 3D culture models, murine models, and canine models. The relevance, strengths, and limitations of each model in OS research are explored. In particular, we highlight a range of potential therapeutics identified through these models. These instances of successful drug development represent promising pathways for personalized OS treatment.

## 1. Introduction

Osteosarcoma (OS), the most common type of primary bone malignancy in childhood and adolescence, comprises approximately 60% of the common histological subtypes of total malignant childhood bone tumors [1]. The five-year survival rate for patients with localized OS is about 60–70%, whereas it is less than 20% for patients with metastatic OSs [2,3,4]. Thus, the treatment of metastatic OS is a significant clinical challenge that requires extensive preclinical studies to discover new therapeutic strategies. The standard of treatment for metastasized OS consists of surgical resection, chemotherapy, and targeted therapy, with the ultimate goal of maximizing tumor shrinkage and arresting further tumor growth and spread, accompanied by locoregional treatment whenever possible [5]. Even though decades of efforts devoted to improving the survival rate of OS patients have brought significant advances, novel therapeutic regimens for this patient population are needed. One of the major obstacles to identifying and developing efficient therapeutic options for the treatment of patients is effectively translating scientific knowledge from bench to bedside [6]. A number of drug candidates initially show success in laboratory models but fail in clinical trials, and many clinical trials have failed due to inappropriate patient selection [7].

The establishment of preclinical models, which faithfully recapitulate OS pathogenesis, represents a key tool for testing novel treatment options that could provide long-term benefits for the treatment of OS patients [8]. Different types of preclinically available models and techniques for modeling disease “at the bench” are used to unravel significant genetic, transcriptomic, and proteomic players taking part in the initiation and progression of the OS, and to include the identification of anticancer agents with improved translational potential, leading to precision medicine [9]. An ideal preclinical OS model should not only show close histological similarity to the tumor of origin and maintain druggable genomic alterations for targeted approaches, but should also address practical issues, such as through easy handling and good in vitro and in vivo growth characteristics [9]. The discovery and testing of novel therapeutic agents have been conducted using in vitro, ex vivo, and in vivo models. Human cancer-derived cell lines have significantly contributed the understanding of cancer biology and key intracellular mechanisms. Three-dimensional cultures, mouse models, and canine models have become a breakthrough for the expansion of vital tissue, which is decisive for applied research and therapeutic studies.

Recently, preclinical models have increasingly found application across various malignancies, such as colorectal cancer, cutaneous melanoma, thyroid cancer, and pancreatic cancer, becoming indispensable tools in drug discovery [10,11,12]. Orphan cancers, particularly OS, benefit from preclinical models to discover anticancer drugs. While there are relevant studies focused on OS drug development, none have provided a comprehensive description of the rapid progress in recent OS drug discoveries from both in vivo and in vitro modeling perspectives [13]. In this review, we aim to discuss both the advantages and challenges of the current experimental preclinical models of OS, along with the inherent limitations of each model. We highlight the most important studies and illustrate how they can be used to address missing gaps within OS cancer research. Lastly, we focus on the translational purpose of individual models and discuss their potential and new directions, eventually leading to personalized medicine as the ultimate goal in molecular oncology.

## 2. Advance in OS Cells and Models

OS is not a uniform mass of cancer cells, but a complex, organ-like structure with diverse cell types influenced by various environmental factors [14,15]. An individual with OS is subject to a multitude of complex biological, structural, mechanical, and soluble factors that may affect the effectiveness of potential therapeutics [7]. Tumor-associated cells typically located in the vicinity of cancer cells include fibroblasts, immune cells, and endothelial cells. Structural factors include the architecture of the tumor itself (three-dimensionality), with the spherical nature of cell-to-cell interactions and the presence of extracellular matrix (ECM) key features. In addition, the mechanical forces applied by the surrounding microenvironment are important to tumor dynamics. Soluble factors may include gradients of chemicals, such as nutrients and gases, e.g., glucose and oxygen. Accordingly, the need for a more comprehensive range of OS models that precisely simulate this multifaceted tumor microenvironment is imperative for propelling advancements in drug discovery. The currently available models, such as traditional cell lines, 3D cultures, murine models (including patient-derived xenograft (PDX) and transgenic models), and canine models, each embody a unique aspect of OS. Collectively, they offer a comprehensive view of OS biology [16]. Each model comes with its distinct advantages and drawbacks (Table 1).

### 2.1. Two-Dimensional (2D) OS Cell Models

Two-dimensional OS cell culture models, frequently used in vitro, have long been a conventional method for studying tumorigenesis, cancer biology, and drug discovery [17]. These basic models not only aid in understanding the molecular and phenotypic characteristics of cells, but also facilitate hypothesis testing for translational research and the creation of genome–drug response correlations [18]. The popularity of established cell lines is attributed to their practicality, cost-effectiveness, and speed in delivering experimental results.

Pioneering research from Mohseny’s laboratory has identified OS cell lines that exhibit key features of tumorigenesis, such as immune attraction (U2OS), angiogenesis (IOR/OS-14 and HOS-143B), the invasion of adjacent tissues (MHM), in vivo differentiation (IOR/OS9), and metastasis (HOS-143B) [19]. These OS cell lines offer a broad range of tumorigenesis attributes, thus accelerating the drug discovery process [19]. For instance, drug response assays with SAOS-2, U2OS, SJSA-1, HOS, and MNNG human OS cell lines have been instrumental in uncovering the therapeutic potential of compounds like afatinib [20]. Afatinib was observed to inhibit OS cell viability, motility, and migration by suppressing the activation of the ErbB pathway [20].

Further research has enriched the variety of available OS cell lines. Thanindratarn and colleagues unveiled a novel recurrent OS cell line, OSA 1777, which provided novel insights into the mechanisms of OS recurrence and metastasis [21]. Similarly, VanCleave and team introduced a unique, enduring human cancer cell line, COS-33, which precisely mirrors the original tumor’s histopathology, cytogenetic intricacy, osteoblastic activity, and drug sensitivity [22]. Notably, VanCleave’s research revealed that this cell line has a particular dependency on the mTOR pathway, a critical regulator of cell growth and proliferation [22]. Such dependency is of high clinical relevance as there are already clinically approved drugs targeting this pathway [22]. Consequently, COS-33 could serve as a new or complementary tool for drug screening, and for further elucidating OS dependencies on key signaling pathways like the mTOR pathway [22].

Proteomic analysis reveals that established OS cell lines can partially depict primary tumors, demonstrating their significant value in illustrating tumor biology [23]. However, these cell lines often exhibit systemic proteomic differences compared to the original tumors, reflecting variations in tumor stroma, extrinsic signaling, and growth conditions [24]. Despite their easy manipulation, adaptability for global studies, and suitability for high-throughput applications, their questionable accuracy in reflecting clinical samples is a persistent concern [25,26].

The 2D cell lines bear inherent limitations, which include genetic homogeneity from in vitro selection, gene drift upon successive passaging, and a deficiency in authentically mimicking interactions between cancer cells and their microenvironment or reproducing patient treatment responses [27,28]. Furthermore, these models fall short in fully capturing the intricacy and pathophysiology of in vivo tumors [29,30,31]. Despite these, 2D models remain essential. Their rich data have propelled the evolution of more advanced in vitro preclinical models and have corroborated previous findings in clinically relevant models.

### 2.2. Three-Dimensional (3D) OS Cell Models

**Figure 1:**
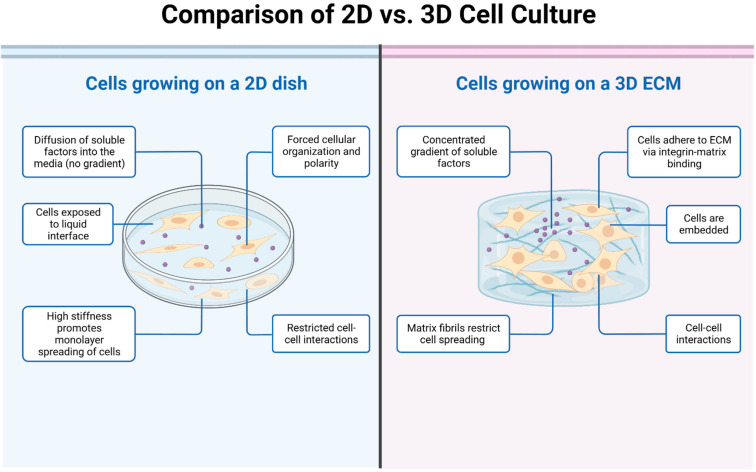
The picture illustrates the key differences between 2D and 3D cell cultures. In the 2D cell culture model, cells are grown and adhered to a flat surface, such as a petri dish or a culture flask. The cells form a monolayer and spread out in a single plane. In the 3D cell culture model, cells are grown in a three-dimensional environment that better mimics the natural tissue architecture. Cells can be encapsulated within hydrogels or scaffolds, allowing them to grow and interact in a more physiologically relevant manner.

Advancements in tissue engineering have led to the development of 3D constructs, such as spheroids and organoids, designed to more accurately replicate the complex intracellular dynamics and microenvironments of OS [32,33]. Spheroids are cellular aggregates embedded with collagen type I, with outer cells adhering to and invading into the matrix [34]. These compact, globular structures can mimic diverse microenvironments within tumors, including anoxic, hypoxic, and oxic niches [35]. Organoids are self-organized three-dimensional structures derived in vitro from pluripotent or adult stem cells [9]. They create a microanatomy that closely resembles native tissue with differentiated cell types and organ-specific compartmentalization [36,37]. These 3D models, with their advanced tissue mimicry, present a promising platform for the advancement of personalized medicine. They can be expanded in vitro and subjected to various drug treatments to determine the most effective therapy for each individual patient. Based on the chosen preparation method, 3D models can be crudely classified into three categories: (i) scaffold-free sphere models, (ii) scaffold-based sphere models, and (iii) organoid models [38,39,40,41] (Table 2 and Figure 1).

#### 2.2.1. Scaffold-Free Sphere Models

**Figure 2:**
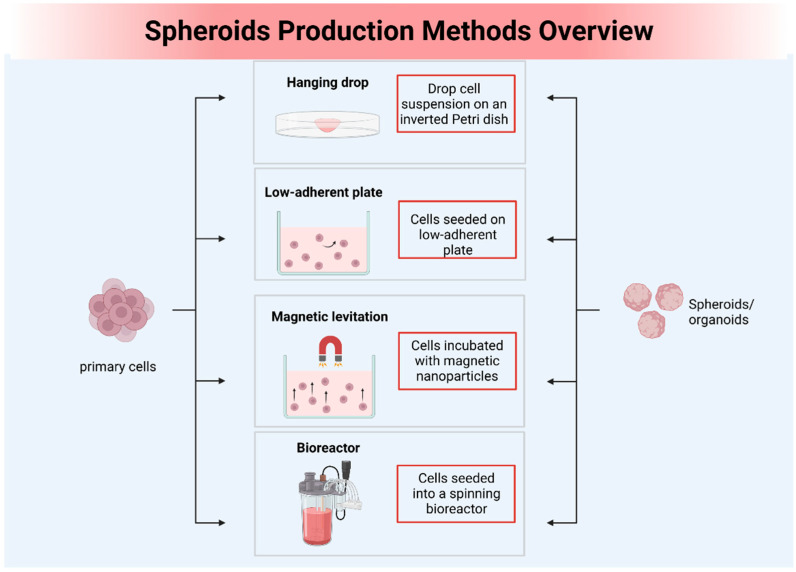
The picture illustrates the preparation methods for spheroids, three-dimensional cellular aggregates.

Scaffold-free spheroid models are 3D cell culture systems where cellular aggregates form in an environment lacking any artificial matrix or scaffold. OS spheroids, serving as a model encompassing the synergistic effects of cell–cell and cell–matrix interactions, have proven useful in enhancing clinical responsiveness to chemotherapy and advancing personalized cancer medicine research [61]. This has been enabled by the utilization of various platforms that support the development of scaffold-free 3D cellular structures, including low-adhesion plates [62], nanoparticle-facilitated magnetic levitation [63], hanging drop plates [64], and rotary cell culture [65] (Figure 2).

Hanging drop methodology has been employed in various studies to generate 3D OS multicellular spheroids, such as MG-63. One such study reported differential anticancer efficacy between 3D and 2D cultures upon treatment with two quinoline–platinum complexes—Pt(Cl)2(quinoline)(dmso) and PtCl(8-O-quinoline)(dmso) [42]. The results illustrated the potential of this approach for toxicity screening studies [42]. In another study, Franceschini et al. utilized the same technique to generate multicellular tumor spheroids from MHM, MG63, and SAOS2 OS cell lines [49]. Their findings linked a low expression of nicotinamide phosphoribosyltransferase (NAMPT) RNA with NAMPT methylation near the transcription start site in both OS cell lines and primary tumors [49]. These data posited NAMPT as a promising therapeutic target for OS, and suggested that low NAPRT expression could serve as a potential biomarker for patient selection [49].

The liquid overlay method offers an alternative strategy for establishing scaffold-free OS models [66]. These techniques aim to prevent cell adhesion to container surfaces, such as low-adhesion plates, by coating them with non-adherent materials like agar or poly-hydroxyethyl methacrylate [67]. An exemplifying study by Ohya et al. utilized this approach to generate MG-63 spheroids [47]. Their research revealed a significant increase in the levels of large-conductance Ca^2+^-activated K^+^ channel K_Ca_1.1 within these spheroids [47]. Furthermore, they found that a K_Ca_1.1 inhibitor effectively countered the chemoresistance of MG-63 and human chondrosarcoma SW-1353 spheroid models to paclitaxel, doxorubicin (DOX), and cisplatin, hinting at a novel therapeutic strategy through K_Ca_1.1 inhibition to sensitize OS cells to chemotherapy [47]. Similarly, Li et al. utilized the liquid overlay method to reveal that a new bisphosphonate-loaded microarc oxidation coated magnesium–strontium alloy pellet can inhibit OS [50]. These pellets impaired the formation of multicellular tumor spheroids by the OS cell line UMR-106 in a 3D cell culture environment [50]. A pioneering study by Baranski et al. used this approach to investigate potential therapies for micro metastatic OS [51]. They examined the effects of ^224^Ra/^212^Pb-TCMC-TP-3 (dual alpha solution) on multicellular spheroids that mimic this disease state [51]. They found that OHS spheroids of 253 ± 98 µm diameter treated with ^212^Pb-TCMC-TP-3 for 24 h disintegrated within 3 weeks [51]. Moreover, both single and dual alpha solutions combined with TP-3 demonstrated enhanced cytotoxicity in spheroids of a clinically relevant size, outperforming rituximab [51].

Single tumor spheroid models may not fully capture the complexities of the tumor microenvironment [68]. To address this limitation, hybrid systems involving different cell types have been utilized for anti-tumor drug evaluations. Pang et al. developed the first co-culture spheroid model for OS, which enables the manipulation of cancer states (early/late) through altering the ratio of stromal to OS cells [52]. Interestingly, this stimulatory effect on stromal cells was abolished when these supplements were combined with chemotherapeutics [52]. This intriguing finding revealed a paradoxical relationship between tumor elimination and bone regeneration, thus contributing to the development of more effective therapeutic strategies for OS [52]. In a separate study, Marshall et al. put forth a nanocarrier delivery platform with improved tumor specificity and penetration in a 3D human MG-63 spheroid model [56]. They employed the double emulsion method to synthesize PEG-PLGA nanoparticles, encapsulating DOX and Na^131^I within the inner core [56]. These were further conjugated with an epidermal growth factor receptor (EGFR) antibody for targeted delivery to human MG-63 cells [56]. This multifunctional I^131^ radio-nanotherapeutic targeting anti-EGFR provides a tailored treatment option for OS, underlining the potential of 3D multicellular spheroid models in anticancer drug discovery and development [56].

Cancer stem cells (CSCs) possess stem cell characteristics and exert a dominant influence on tumor initiation, dormancy, recurrence, and metastasis [69]. In a noteworthy investigation, Ozturk et al. isolated CSCs from the SAOS-2 cell line, using agar molds to construct a scaffold-free 3D model, termed as a ‘tumoroid’ [44]. This 3D environment was found to maintain the stem cell phenotype for a longer duration compared to conventional two-dimensional (2D) cultures, thereby enhancing the relevance of screening, and improving targeting efficiency during pharmaceutical testing [44]. Additionally, Cortini et al. developed 3D OS spheroids that mimic not just the oncogenesis and cellular proliferation processes, but also the complex cell ECM interactions [60]. These 3D OS spheroids, composed of metastatic or non-metastatic OS cells and mesenchymal stromal cells (MSCs), displayed ECM protein deposition (including Type I collagen, Type III collagen, and fibronectin) at the interface between tumor cells and MSCs [60]. Their research underscored that ECM protein deposition plays a crucial role in evaluating drug response, suggesting that targeting these proteins could potentially improve outcomes in chemoresistant tumors [60].

#### 2.2.2. Scaffold-Based Sphere Models

Scaffolds provide a 3D structure that supports the adhesion and proliferation of tumor cells, facilitating the formation of spheroids within their interstices [70]. These scaffold-based culture models have proven particularly useful in studies of OS, as they can mimic the complex microenvironment of bone tissue. Notably, both natural and synthetic scaffolds exhibit macro- and microstructural configurations that closely resemble trabecular bone, making them ideal for investigating bone mineralization processes [71,72,73]. Specifically, type I collagen, the principal constituent of bone tissue, is extensively used as a 3D scaffold to encourage the expansion of OS cell lines and promote bone mineralization [74]. Hydroxyapatite (HA), a naturally occurring mineral form of calcium apatite known for its excellent biocompatibility, is an optimal candidate for bone repair and substitution [75]. In a recent study, González Díaz et al. developed an OS model using micro-ribbon scaffolds with bone-mimicking compositions [54]. The team fabricated gelatin micro-ribbon scaffolds both with and without HA nanoparticles to simulate the two primary constituents of bone matrix: type I collagen and minerals [54]. When testing the dose response to doxorubicin, the 3D micro-ribbon models were found to maintain OS drug resistance phenotypes more effectively than 2D cultures [54]. Moreover, it was observed that bone mineralization further enhances drug resistance in OS [54]. Using a similar approach, Tornín et al. developed Collagen Type 1 (Col1)/HA Nanoparticles (nHA)-FITC bone-like scaffolds [58]. They demonstrated that cold plasma treatment could selectively target tumorigenicity, and inhibiting the STAT3 signaling pathway significantly reduced the tumorigenicity and survival of OS cells [58]. These findings suggest potential strategies for enhancing therapeutic approaches in OS treatment [58].

Although collagen and HA are commonly used in the fabrication of 3D scaffold models for OS [76], most of these models fail to simultaneously incorporate a scaffold and a biomimetic matrix, both of which are crucial in accurately simulating tumor cell behavior [76]. Pavlou M. et al. first proposed a scaffold-based geometrically compartmentalized 3D model of OS, which was composed of a core cellular artificial cancer mass and a surrounding acellular ECM compartment [43]. The 3D model matrix was enriched with bone marrow proteins including laminin, fibronectin, and NuOss^®^ bone granules, aiming to investigate the impact of a biomimetic matrix on OS cell behavior [43]. An analysis of the DOX-treated model revealed that OS cells grown in a complex matrix composition exhibited a greater degree of change in metabolic activity than other basic tumoroids when exposed to DOX [43]. It suggested that the 3D culture model developed in this study more closely resembles the in vivo situation compared to previously established models [43]. Following this, Pierrevelcin et al. created a novel 3D model that takes into account the complexity of bone structure and its extracellular matrix, as well as the presence of macrophages, a hypoxic microenvironment, and tumor cells [55]. They built a 3D bone model by combining a physiologically relevant matrix containing collagen and chitosan, which was then cultured with OS cells and M2 macrophages under hypoxic conditions [55]. They further validated the model’s anticancer efficacy by testing the feasibility of cabozantinib and rapamycin [55]. Their results showed that incorporating hypoxic features and M2 macrophages is essential for simulating intercellular pathophysiological interactions, as they influence OS cell behavior and introduce extrinsic heterogeneity [55]. This can ultimately impact responses to therapies [55].

Building on the use of diverse scaffold materials, the hydrogel scaffold has shown great potential as a platform for OS modeling [33]. Monteiro et al. developed cellular complexes containing human bone marrow mesenchymal stem cells and fetal human osteoblasts by using a co-culture humanized 3D OS model with the scaffold of methacryloyl platelet lysates-based hydrogels [48]. They found a positive outcome with synergistic tumor–stromal cell interaction in OS tumors in terms of growth, invasive ability, and improved resistance to DOX treatment [48]. This highlighted the potential of the herein established co-culture model as a reliable platform for drug screening [48]. Lin et al. fabricated a 3D bio-printed OS model (3DBPO) that contains OS cells and a shrouding ECM analogue composed of gelatine methacrylamide (GelMA) and hyaluronic acid methacrylate in a 3D frame [57]. They confirmed that 3DBPO models exhibited autophagy levels closer to those in vivo compared to conventional in vitro 2D and CSC models [57]. Furthermore, the results obtained using 3DBPO models are also consistent with the clinical drug screening results [57]. Similarly, He et al. (2022) utilized microfluidic technology to construct honeycomb-like porous GelMA hydrogel microspheres for OS cell culture [59]. They showed that 3D structural microspheres are capable of maintaining the biological properties and tumorigenicity of OS cells to a greater extent [59].

Further expanding the variety of scaffold types used in 3D OS models is crucial for advancing drug development. Contessi Negrini and colleagues (2022) employed a 3D-printed polyurethane scaffold and in vitro generated bone extracellular matrix to establish an OS model that closely mimics the tumor microenvironment [53]. Their model successfully simulated the complex mechanical and biochemical interactions inherent in the bone tumor microenvironment [53]. The study emphasized the potential of scaffold-based models in providing more accurate platforms for drug screening and for understanding the OS microenvironment.

#### 2.2.3. Organoid Models

Due to uncertainty in the growth factors required for some tumor tissues, it is difficult for the corresponding organoids to grow in vitro for a long time [77]. At present, tumor organoids are mainly derived from epithelial tumors, and methods for generating nonepithelial cell-derived organoids (such as OS) still need further research [78]. A significant exception was presented by He et al. in 2020, who successfully established a patient-derived organoid culture system modeling lung metastatic OS [79]. This system effectively mimicked the complex tumor microenvironment while maintaining the histological and molecular attributes of the original tumor [79]. Displaying considerable promise, this organoid culture system offers a robust platform for precision medicine, with potential applications in anti-tumor drug screening, immunotherapy assessment, and broader preclinical investigations for mesenchymal-originating cancers [79]. By utilizing organoid models, researchers can comprehensively assess the efficacy and toxicity of potential drug candidates, leading to the identification of novel therapeutic targets and the design of personalized treatment strategies [80]. This underscores the pressing need for further exploration into the complexity of organoid models and their implications for OS drug development.

### 2.3. Murine Models

**Figure 3:**
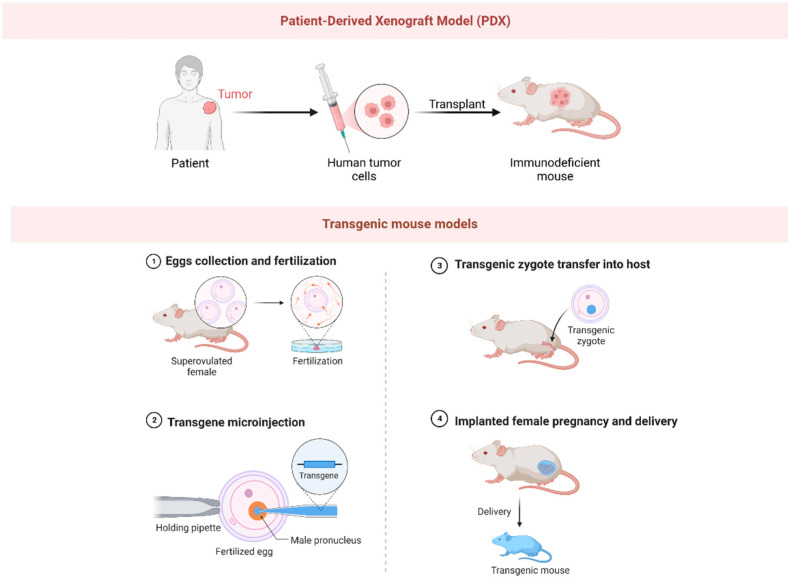
The picture illustrates the preparation methods for PDX (patient-derived xenograft) model and transgenic mouse model.

OS murine models include xenografts and genetically engineered models. Xenograft models are characterized by the implantation of patient-derived OS cell lines into immunodeficient mice [8]. This model maintains the heterogeneity of human tumors, providing an advantageous platform for the evaluation of therapeutic efficacy and the study of tumor–host interactions [8]. Conversely, genetically engineered models, often utilizing specific oncogene alterations, present an ideal system for studying OS pathogenesis and progression [81,82]. Xenograft and transgenic mouse models have emerged as indispensable experimental systems, demonstrating exceptional proficiency in accurately replicating the intricate characteristics of OS in vivo [83,84] (Figure 3).

#### 2.3.1. Xenograft Mouse Models

Xenograft models are predominantly categorized into two types: direct xenograft models (DXM) and cell-line-derived xenograft models [85]. Currently, the PDX model, a specific type of DXM, is more extensively utilized in the investigation of OS therapeutics [86]. Historically, the optimization of standard chemotherapeutic drugs, such as cisplatin, DOX, ifosfomide, and methotrexate, has been achieved through the utilization of PDX OS models [87]. In recent years, an array of over 100 compounds has been subjected to rigorous screening via PDX models to ascertain their therapeutic efficacy against OS [88]. One exemplary example is anticarin-β, a naturally derived coumarin compound extracted from the bark of Antiaris toxicaria Lesch [89]. Utilizing tumor tissues procured from OS patients, researchers successfully established PDX models via subcutaneous transplantation into immunodeficient mice [89]. The clinical potential of anticarin-β was subsequently evaluated utilizing these mouse PDX models [89]. Remarkably, anticarin-β demonstrated potent inhibitory impacts across diverse stages of OS, notably including lung metastasis, in the PDX models [89]. These promising outcomes suggest that anticarin-β may offer a viable therapeutic strategy for the management of OS, particularly in the context of advanced or metastatic cases [89].

Despite bearing identical genomic modifications to their corresponding human tumors, PDX models inherently present certain constraints. In particular, the therapeutic response observed within these models does not invariably imply successful clinical trial efficacy [83]. For instance, glembatumumab vedotin, an antibody–drug conjugate (ADC), and eribulin, a microtubule inhibitor, showed potential against OS in PDX models [90]. However, their actual effectiveness in patients suffering from recurrent OS was found to be decidedly limited [90,91]. In the case of eribulin, the observed discrepancy likely stems from a failure to adequately consider the pharmacokinetic variations between mice and humans [92]. One significant limitation is that PDX tumors must be implanted in immunodeficient mice, which results in these models falling short of reproducing the immunological intricacies of cancers and their treatments. This limitation is particularly noticeable when assessing the effectiveness of immunotherapies. Determining how activity levels in PDX models translate into clinical efficacy presents another challenge. The evaluation could be based on either the percentage of models demonstrating a response, or the intensity of the response within an individual model. Evaluating the predictive value of these preclinical models is complicated, particularly with the scarcity of novel agents that exhibit clinical activity, thereby constraining the derivation of reliable insights from these models.

#### 2.3.2. Transgenic Mouse Models

Besides PDX models, various transgenic OS models have been developed, and yet their application in drug discovery remains notably infrequent [8]. For example, Nannan et al. crafted a unique transgenic mouse model, wherein tumor protein p53 was specifically inactivated in osteoblasts [93]. The study’s results revealed that inactivating p53 within osteoblasts led to an increase in local bone formation [93]. This suggested a previously unexplored role for p53 within these cells, positioning it as a potential regulator of bone metabolism [93]. The authors’ novel findings have critical implications for devising therapies for diseases with abnormal bone activity, such as osteoporosis and OS [93]. Wang et al. delved into the intricate relationship between the S-phase kinase-associated protein 2 (SKP2) and cyclin-dependent kinase inhibitor 1B (p27) [94]. Their groundbreaking study used a mouse model with Rb1 and Trp53 double knockouts within osteoblastic lineage cells [94]. This investigation highlighted the profound effect of the SKP2-p27 interaction on OS’s progression and stemness [94]. Their discovery suggests potential novel targets for therapeutic intervention, thereby expanding our understanding of OS’s complex molecular pathways [94]. In a pivotal study, Ferrena et al. utilized mouse models deficient in Retinoblastoma 1 (Rb1) and Tumor Protein p53—two key genes in OS—to examine the effects of SKP2 knockout [95]. Their results revealed that SKP2 deficiency induced significant immune infiltration within the tumor microenvironment, suggesting a potential immune response against OS [95]. Further, the SKP2 knockout triggered a transcriptional program associated with a favorable prognosis [95]. This crucial work, leveraging interactions within the tumor microenvironment, paves the way for novel osteosarcoma treatment strategies [95]. These transgenic models provide the opportunity to assess OS within their native microenvironment, thereby addressing certain limitations associated with PDX models. Nonetheless, due to the dissimilarities between murine and human immune systems, transgenic models may not fully replicate immune reactions to OS in patients. To overcome this limitation, researchers have begun developing ‘humanized’ mouse models—PDX models of OS in immunocompromised mice reconstituted with human immune cells [96]. However, this research field remains in its infancy, with relatively few models currently available. Nonetheless, it represents a promising frontier for osteosarcoma research, with the potential to revolutionize our understanding of the disease and our approach to its treatment.

### 2.4. Canine Models

Dogs represent a highly relevant model for studying human OS due to several compelling parallels. Just as in humans, OS is the most prevalent bone cancer in dogs, frequently manifesting in the long bones—a clinical feature consistently observed in both species [97]. Furthermore, the clinical intervention process for osteosarcoma, which encompasses preoperative to postoperative procedures, exhibits a striking resemblance between canines and humans. This parallelism highlights the significance of the canine model in enhancing the comprehension of osteosarcoma, and in the development of therapeutic approaches [97]. A unique aspect that highlights the relevance of the canine model is that, apart from humans, dogs are the only mammals known to spontaneously develop OS within the context of an intact immune system [98]. These marked similarities not only highlight the dog as a powerful model for understanding the biology and clinical progression of OS, but also emphasize its potential in advancing novel therapeutic approaches for OS.

Recent investigations employing the canine model have opened promising pathways for the development of innovative pharmaceutical treatments in OS. Canine OS cell lines have proven to be a vital resource in the field of drug discovery. In their research, Chirio et al. used these cell lines to evaluate how well DOX-loaded, calcium phosphate-coated lipid nanoparticles worked. Their laboratory results highlighted the promise of these particles in overcoming drug resistance and boosting the effects of chemotherapy [99]. Similarly, Yang et al. investigated the synergistic effects of sorafenib and DOX in both human and canine OS cell lines [100]. Their findings revealed that the combination of these two drugs exhibited enhanced efficacy in inhibiting cell proliferation, reducing migration and invasion abilities, and inducing cell cycle arrest [100]. The in vivo canine OS model provides a valuable tool for studying drug behavior within a complex physiological context. A study by Regan et al. investigated the efficacy of losartan, a drug commonly used to treat hypertension, in combination with the kinase inhibitor toceranib, in the treatment of metastatic OS in 28 dogs [101]. They demonstrated that losartan effectively blocked the recruitment of monocytes elicited by OS, and, when combined with toceranib, resulted in significant clinical benefits in dogs with metastatic OS [101]. These results hold significant implications for OS drug development, suggesting a potential therapeutic strategy that could improve treatment outcomes for both human and canine patients.

However, it is imperative to acknowledge certain limitations associated with using the canine OS model for drug development. Firstly, significant differences might exist between canines and humans in the pharmacokinetic and pharmacodynamic profiles of drugs due to species-specific metabolic processes [102]. This could potentially create discrepancies in drug efficacy and safety assessments [102]. Additionally, ethical considerations concerning animal welfare in experimental settings must be strictly addressed, which may limit the scope and application of certain investigational procedures [103]. Thus, while the canine model provides crucial insights for OS drug development, it is essential to balance its use with complementary models and strategies to ensure comprehensive and accurate results.

## 3. Conclusions and Future Perspectives

The overarching aim within the OS field is to develop effective therapeutic strategies, providing a cure for every patient with OS. Consequently, it is imperative for researchers to refine OS models that authentically replicate the development, heterogeneity, plasticity, progression, and unique molecular characteristics of human OS. This review encapsulates the current state of OS drug development and emphasizes the pivotal role of a variety of preclinical models, both in vitro and in vivo. Each model, with its unique advantages and inherent limitations, contributes to a versatile toolset, thereby facilitating drug discovery, validating therapeutic efficacy, and informing personalized treatment strategies for OS. Cell lines are instrumental for OS drug development, providing a controlled environment for the rapid assessment of drug effects and toxicities. Among 3D culture models, scaffold-free spheres offer a more physiologically relevant representation of human microenvironments, thereby enhancing the investigation of OS’s intricate biological properties. Scaffold-based spheres further the clinical translation by simulating the structural characteristics of human OS tissues. Organoids, replicating the complexity and heterogeneity of human OS, signify a substantial advancement in in vitro modeling. In the context of animal-based studies, murine xenografts and genetically engineered models offer realistic biological contexts for OS research, holding reliability in drug screening and translational applicability. Canine models, due to their biological resemblance to human OS, provide insights into human OS progression, narrowing the translational gap. Collectively, these models contribute to a comprehensive toolset, thereby facilitating OS drug discovery and personalized treatment strategies.

In terms of future research perspectives, there is a clear necessity for the development and validation of enhanced, representative models of OS that closely simulate the intricate tumor microenvironment and immune interactions. Future research focusing on OS drug development models might consider the following aspects: (1) augmenting model sophistication: strengthening the biological fidelity of OS models, especially with respect to simulating the intricate tumor microenvironment and immune interactions; (2) utilizing innovative techniques: implementing advanced techniques like CRISPR/Cas9 gene editing and single-cell sequencing to better delineate the molecular intricacies of OS; (3) expanding immunotherapy investigations: extending the exploration of immunotherapies, given their promise in other cancer types yet relative under-exploitation in OS research; (4) developing more robust models: enhancing the robustness of OS models, particularly through the generation of humanized mouse models and sophisticated organoid models; (5) encouraging collaborative efforts: fostering collaborative efforts across various research domains and clinical practice, and leveraging ongoing technological advancements to expedite the discovery of effective OS therapeutics. These integrative approaches can provide a comprehensive view of OS, paving the way for significant breakthroughs in its treatment.

## Figures and Tables

**Table 1:** Overview of the advantages and disadvantages of in vitro and in vivo osteosarcoma models.

Model Type	Model	Advantages	Disadvantages	Translational Potential
In vitro	Cell lines	Easy to culture and maintain. High reproducibility across experiments. Cost-effective for large-scale screening.	Limited representation of tumor heterogeneity. Lack of interaction with the tumor microenvironment. Potential genetic drift with prolonged culture. Poor recapitulation of clinical response.	Rapid platform for drug screening. Quick evaluation of drug effects. May lack human-like environment.
	Sphere models	Mimic 3D tumor architecture. Promote cell–cell interactions. Better represent tumor heterogeneity. Useful for studying cancer stem cells.	Difficult to standardize and control size. Lack of in vivo microenvironment. Limited scalability for drug testing. Lower throughput compared to 2D cultures.	Resemble human microenvironments. Accurate in drug sensitivity study. Complex cultivation. Low reproducibility.
	Organoids	Capture both tumor cells and microenvironment. High physiological relevance. Enable study of organ-specific interactions. Useful for personalized medicine studies.	Technically challenging to establish and maintain. Time-consuming and expensive. Difficult to scale-up for high-throughput screening. Depend on the availability of patient-derived tissues.	Similar to human OS structure. Simulate human OS complexity. Complex production. Inter-sample variability.
In vivo	Xenograft mouse models	Preserves tumor heterogeneity. Predicts clinical response.	Time-consuming and costly. Infeasible for large-scale studies. Involves complex ethical issues. Lacks human immune microenvironment.	Study OS in human-like context. Reliable in drug screening and toxicity assessment. Facilitate translational process. May not replicate all human tumor characteristics.
	Transgenic mouse models	Genetically defined model. Recapitulate tumor progression in a controlled manner. Enables the study of oncogenesis and tumor progression. Useful for preclinical validation of target genes.	Complex and time-consuming to generate. May not fully represent human disease. Costly to maintain. Possibility of non-physiological overexpression or deletion of genes.	Mimic human OS genetics and biology. Allow manipulation of specific genes. Time-consuming to develop. Expensive and complex maintenance.
	Canine models	Naturally occurring osteosarcoma with intact immune system. Similar clinical presentation and interventions to humans. Large size facilitates serial biopsies and imaging. Useful for translational research and comparative oncology.	Genetic and environmental diversity. Longer lifespan extends study duration. Ethical considerations of animal use.	Similar to human OS biologically. Critical in translational research. Ethical considerations. High costs.

**Table 2:** Three-dimensional in vitro models for osteosarcoma and drug discovery research.

Year	Method	Technique	Material/Technique	Cell Line	Pharmaceutical/Therapeutic	Ref.
2019	Spheroids cultures	Scaffold-free	Hanging drop technique	MG-63	PtCl(8-O-quinoline)(dmso) (2)	[42]
2019	Spheroids cultures	Scaffold	High density collagen	MG-63; 148B;	Biomimetic matrix	[43]
2020	Spheroids cultures	Scaffold-free	Liquid-overlay	SAOS-2	CSCs tumoroid	[44]
2020	Spheroids cultures	Not Mentioned	Not Mentioned	U2OS; MG-63;	Gamabufotalin (GBT)	[45]
2020	Spheroids cultures	Not Mentioned	Not Mentioned	U2OS;	Novel imidazopyrimidine derivatives	[46]
2021	Spheroids cultures	Scaffold-free	Liquid-overlay	MG-63 SW-1353	Ca^2+^-activated K^+^ channel K_Ca_1.1	[47]
2021	Spheroids cultures	Scaffold	PLMA-based hydrogels	hBM-MSCs; MG-63	A co-culture model for drug screening purposes	[48]
2021	Spheroids cultures	Scaffold-free	Hanging drop technique	MHM; MG63; SAOS-2	Targeting NAMPT	[49]
2021	Spheroids cultures	Scaffold-free	Liquid-overlay	UMR-106	BP-loaded MAO-coated Mg–Sr alloy pellet	[50]
2022	Spheroids cultures	Scaffold-free	Liquid-overlay	OHS	^224^Ra/^212^Pb-TCMC-TP-3	[51]
2022	Spheroids cultures	Scaffold-free	Liquid-overlay	SaOS2	A novel model for early and late-stage osteosarcoma.	[52]
2022	Spheroids cultures	Scaffold	Polyurethane	SAOS-2	Assess new treatments.	[53]
2022	Spheroids cultures	Scaffold	Gelatin and hydroxyapatite	MG-63	The 3D GelHA models can predict the in vivo efficacy of drug targets	[54]
2022	Spheroids cultures	Scaffold	Collagen and chitosan	OSL08; OSL16; OSL20	Reconstructed high-grade osteosarcoma and its immune and extracellular matrix microenvironment	[55]
2022	Spheroids cultures	Scaffold-free	Liquid-overlay	MG-63	I-131 radio-nanotherapeutic	[56]
2022	Spheroids cultures	Scaffold	GelMA/HAMA hydrogel.	HOS; 143B; U2-OS cells	Autophagy-targeted therapy	[57]
2022	Spheroids cultures	Scaffold	Sponge-like Col1/hydroxyapatite nHA	SaOS-2; G-292; U2 OS	Cold atmospheric plasmas and PTL	[58]
2022	Spheroids cultures	Scaffold	Honeycomb-like GelMA hydrogel	K7M2	Maintain tumorigenicity preferably.	[59]
2023	Spheroids cultures	Scaffold-free	Liquid-overlay	143B; MG63; Saos-2	Targeting ECM proteins.	[60]

Abbreviation: PLMA, meth acryloyl platelet lysates; GelMA, gelatine ethacrylamide; HAMA, hyaluronic acid methacrylate; nHA, nanoparticles; BP, bisphosphonate; MAO, microarc oxidation; Mg–Sr, magnesium–strontium; PTL, plasma-treated liquids.

## Data Availability

This study did not generate or analyze new data, rendering data sharing inapplicable.
